# High-intensity interval training accelerates oxygen uptake kinetics and improves exercise tolerance for individuals with cystic fibrosis

**DOI:** 10.1186/s13102-020-0159-z

**Published:** 2020-04-13

**Authors:** Ronen Reuveny, Fred J. DiMenna, Cedric Gunaratnam, Avigdor D. Arad, Gerry N. McElvaney, Davide Susta, Michael Peled, Niall M. Moyna

**Affiliations:** 1 Centre for Preventive Medicine, School of Health and Human Performance, Dublin City University, Dublin, Ireland; 2 Pulmonary Institute, Sheba Medical Center, Tel-HaShomer, Ramat Gan, affiliated with Sackler School of Medicine, Tel Aviv University, Tel Aviv, Israel; 3 Department of Medicine, Division of Endocrinology, Diabetes and Bone Disease, Mt. Sinai St. Luke’s Hospital, New York, USA; 4 Department of Biobehavioral Sciences, Teachers College, Columbia University, 525 W. 120th Street, New York, N.Y 10027 USA; 5 Department of Medicine, Respiratory Research Division, Royal College of Surgeons in Ireland, Education and Research Centre, Beaumont Hospital, Dublin 9, Ireland

**Keywords:** Cystic fibrosis high-intensity interval training oxygen supplementation∙V̇O_2_ kinetics functional capacity exercise tolerance

## Abstract

**Background:**

Exercise training provides benefits for individuals with cystic fibrosis; however, the optimal program is unclear. High-intensity interval training is safe and effective for improving ‘functional capacity’ in these individuals with peak rate of O_2_ uptake typically referenced. The ability to adjust submaximal rate of oxygen uptake (V̇O_2_ kinetics) might be more important for everyday function because maximal efforts are usually not undertaken. Moreover, the ability of high-intensity training to accelerate V̇O_2_ kinetics for individuals with cystic fibrosis could be enhanced with O_2_ supplementation during training.

**Methods:**

Nine individuals with cystic fibrosis completed incremental cycling to limit of tolerance followed by 8 weeks of high-intensity interval cycling (2 sessions per week x ~ 45 min per session) either with (*n* = 5; O2+) or without (AMB) oxygen supplementation (100%). Each session involved work intervals at 70% of peak work rate followed by 60 s of recovery at 35%. For progression, duration of work intervals was increased according to participant tolerance.

**Results:**

Both groups experienced a significant increase in work-interval duration over the course of the intervention (O2+, 1736 ± 141 *v*. 700 ± 154 s; AMB, 1463 ± 598 *v*. 953 ± 253 s; *P* = 0.000); however, the increase experienced by O2+ was greater (*P* = 0.027). During low-intensity constant-work-rate cycling, the V̇O_2_ mean response time was shortened post compared to pre training (O2+, 34 ± 11 *v*. 44 ± 9 s; AMB, 39 ± 14 *v*. 45 ± 17 s; *P* = 0.000) while during high-intensity constant-work-rate cycling, time to exhaustion was increased (O2+, 1628 ± 163 *v*. 705 ± 133 s; AMB, 1073 ± 633 *v*. 690 ± 348 s; *P* = 0.002) and blood [lactate] response was decreased (O2+, 4.5 ± 0.9 *v*. 6.3 ± 1.4 mmol^.^ L^− 1^; AMB, 4.5 ± 0.6 *v*. 5.2 ± 1.4 mmol^.^ L^− 1^; *P* = 0.003). These positive adaptations were similar regardless of gas inspiration during training.

**Conclusion:**

Eight weeks of high-intensity interval training for patients with cystic fibrosis accelerated V̇O_2_ kinetics and increased time to exhaustion. This provides some evidence that these patients may benefit from this type of exercise.

**Trial registration:**

This study was retrospectively registered in the ISRTCN registry on 22/06/2019 (#ISRCTN13864650).

## Background

Recent evidence confirms that exercise training provides myriad benefits for individuals with cystic fibrosis (CF) [1–4]. However, the mode (e.g., aerobic, strength, inspiratory muscle or some combination thereof), frequency, duration and intensity of training to optimise benefit have yet to be clarified [5]. In addition to differences related to subject characteristics (e.g., age, sex, nutritional status, degree of pulmonary impairment and/or inflammation/infection status) [6], this ambiguity likely reflects the fact that different outcome measures have been used to quantify exercise’s beneficial effects. Interestingly, in addition to pulmonary function (e.g., FEV_1_) and health-related quality-of-life estimates, the peak rate of oxygen (O_2_) uptake (V̇O_2_) achieved on a maximal incremental exercise test (V̇O_2peak_) has been routinely used to quantify ‘functional capacity’ [3, 7–10] for individuals with CF. However, while inversely related to hospitlalzation [11] and mortality [12], the degree to which V̇O_2peak_ reflects the capacity for satisfying the demands of everyday function for these individuals has been questioned [13]. Instead, the capacity to rapidly adjust V̇O_2_ in response to submaximal energetic transitions like those encountered on a daily basis (i.e., ‘V̇O_2_ kinetics’) might provide a better indication of functional capacity for these individuals [13]. Faster V̇O_2_ kinetics minimises the size of the ‘O_2_ deficit’ thereby reducing substrate-level phosphorylation and improving the ability to tolerate physical exertion [14]. This has resonance for individuals with CF who typically demonstrate low tolerance for physical activity [15–17]. It is, therefore, not surprising that a strong correlation exists between the V̇O_2_ time constant (a parameter that quantifies the rapidity of the V̇O_2_ response) and disease severity in subjects with CF [18]. With this in mind, it stands to reason that a training program that improves the capacity to rapidly adjust V̇O_2_ in response to lower-intensity energetic transitions like those encountered on a daily basis would likely have clinical implications [13]. An improved ability to sustain higher-intensity exercise (i.e., work performed at a work rate that exceeds those that are associated with physical activity performed during daily living) might also have implications for pulmonary patients with respect to health-related quality of life and physical-activity levels [19].

The exercise intolerance demonstrated by individuals with CF is multifactorial with mechanistic bases that shift as the individual ages and the severity of the disease increases [20]. With respect to oxidative capacity, limiting factors can include an impaired ability to deliver O_2_ to skeletal muscle due to a disease-related decline in pulmonary [20–22] and/or cardiac [23] function (central limitation) and/or a reduced capacity for skeletal-muscle mitochondria to extract/use O_2_ that is delivered (peripheral limitation) [13, 24, 25]. A slower V̇O_2_ response has been observed for CF subjects [13, 18, 25, 26] with the decrement found to be mechanistically linked to impaired muscle extraction and utilization [13]. If an O_2_-utilization limitation to V̇O_2_ kinetics is responsible for the reduced tolerance to exercise displayed by individuals with CF, exercise training to improve mitochondrial function might be particularly important for these individuals.

Recent research confirms that high-intensity interval training (i.e., periods of high-intensity ‘work’ intervals interspersed with recovery intervals of low-intensity exercise or rest; HIIT) provides a potent stimulus for enhancing mitochondrial function [27] and HIIT has proven both safe and effective for improving exercise capacity for individuals with CF [28]. Collectively, this implies that HIIT might be an ideal approach for improving functional capacity in these individuals. However, it stands to reason that to provide maximal peripheral stimulation of muscle mitochondria with HIIT, it is necessary to circumvent any central limitation in pulmonary and/or cardiovascular function that might concurrently be in effect. Oxygen supplementation has proven effective for reducing O_2_ desaturation and decreasing ventilatory and cardiovascular work for CF subjects during incremental maximal [29, 30] and constant-work-rate (CWR) submaximal exercise [29, 31]. Furthermore, hyperoxic inspiration would help to counter the hypoxia associated with high-intensity exercise that influences O_2_-mediated signaling cascades (e.g., HIF1α) which affect the adaptive response to training. Collectively, these alterations consequent to hyperoxic inspiration raise the intriguing possibility that a training regimen involving HIIT performed in conjunction with O_2_ supplementation might be more effective for improving maximal and submaximal exercise capacity in CF subjects compared to HIIT while breathing ambient air.

The main purpose of this study was to assess the beneficial effects of an eight-week HIIT cycling intervention for individuals with CF. Specifically, we made pre-/post-training comparisons for: 1.) the ability of oxidative metabolism to adapt to an increase in external work performed (as indicated by the V̇O_2_ mean response time; MRT); and 2.) the ability to sustain exhaustive exercise (as indicated by the time to limit of tolerance; T_lim_). We hypothesized that: 1.) HIIT would reduce the V̇O_2_ MRT during lower-intensity CWR exercise; 2.) HIIT would increase the time to limit of tolerance during higher-intensity CWR exercise.

## Methods

This study employed a randomized, single-blind design. Participants were randomly assigned to a group performing HIIT with O_2_ supplementation (O2+; *n* = 6) or a group performing HIIT while breathing ambient air (AMB; *n* = 5). Participants blinded to the treatment performed HIIT on a cycle ergometer 2 days per week for 8 weeks. Each exercise session was ~ 45 min in duration. This volume (e.g., weeks of training completed and duration of training sessions) was chosen based on our observations regarding what represents a reasonable time investment for this type of individual. Participants fasted for 4 h and abstained from alcohol for 24 h prior to each exercise session. They also refrained from performing strenuous physical activity for 24 h prior to each visit.

Participants visited the Cardiovascular Research Unit at Dublin City University on three separate days before and after the training program. Each visit was separated by ≥72 h. During these visits, participants performed the pre- and post-training evaluations while in stable health and while not receiving antibiotics or other medications. During the first visit, anthropometric measurements were taken, spirometry and single-breath carbon monoxide diffusion capacity (DLCO) were determined and V̇O_2peak_ and peak rate of minute ventilation (V̇_Epeak_) were measured during an incremental cycling bout. During the second and third visits, participants performed CWR cycling bouts at 30% (CWR_30_) and 70% (CWR_70_) of the peak work rate achieved on the incremental test.

### Subjects

Eleven CF subjects (male, *n* = 6) were recruited to take part in this study. The subjects were recruited from the pulmonary department of a local hospital. All patients had documented and proven CF as indicated by clinical characteristics and identified cystic fibrosis transmembrane conductance regulator (CFTR) alteration mutation and/or pathological sweat chloride test (>60 mmol∙L^− 1^). Participants were eligible for inclusion if they possessed no other diseases that could limit their exercise capacity. Participants were excluded from the study if their forced expiratory volume in 1 s (FEV_1_) was less than 30% of the predicted value and they were not clinically stable. The nature and risks of the study were explained. A plain language statement was read and written informed consent was obtained in accordance with the Hospital Medical Ethics Committee. This research was approved by the Ethics (Medical Research) Committee at Beaumont Hospital in Dublin (#07/83).

### Anthropometric measurements

Height and body mass were measured and double thickness subcutaneous adipose tissue was determined on the right side of the body using a skinfold caliper (Harpenden, Cambridge Scientific Industries, MD, USA). Percent body fat was calculated based on the equation proposed by Jackson and Pollock [32].

### Pulmonary-function tests

Standard pulmonary-function tests including spirometry and measurements of DLCO (Sensormedics Vmax 229, Sensormedics Corp, CA, USA) were undertaken and results were compared to normative values [33, 34]. Predicted maximal voluntary ventilation (MVV) was calculated by multiplying FEV_1_ by 40 [35].

### Incremental cycling test

Participants performed an incremental symptom-limited peak exercise test on an electronically-braked cycle ergometer (Ergoselect 100, Ergoline GmbH) while breathing through a full face mask. Prior to work-rate incrementation, 3 min of gas-exchange data were collected both at rest and during ‘unloaded’ cycling. Importantly, these incremental tests were individualised by setting the rate of incrementation in accordance with the disease severity and fitness level of the participant. For example, if FEV_1_ was less than 40% of the predicted value, an incrementation rate of 5 W∙min^− 1^ was employed. When FEV_1_ fell between 40 and 60% of the predicted value, incrementation was 10 W∙min^− 1^ and for values above 60%, a rate of 10–25 W∙min^− 1^ was used. The goal was to have participants reach their limit of exercise tolerance within 8–12 min. The V̇O_2peak_ was defined as the highest 30-s rolling-average value present during the test. Criteria for maximal effort or symptom limitation on the exercise test were RER >1.15 and/or BR <11 l^.^min^− 1^. Dyspnea and leg fatigue were scored on a Borg scale (0–10) every minute during exercise.

### Constant-work-rate cycling tests

Participants performed two CWR cycling bouts on the same ergometer that was used for the incremental test. These bouts were separated by 2–3 days. As previously mentioned, the work rates for these bouts were set at a percentage of the peak work rate that was achieved on the incremental test; specifically, 30 and 70% for CWR_30_ and CWR_70_, respectively. Hence, these constant-work-rate tests were individualised for each participant based on their ability to perform incremental work at a maximal level. Participants performed CWR_30_ for 10 min to provide an adequate amount of time to determine V̇O_2_ kinetics whereas CWR_70_ was continued for 30 min or until limit of tolerance if it occurred prior to assess exercise endurance. Both bouts were preceded by 10 min of unloaded cycling. Gas-exchange, heart rate (HR) and peripheral capillary O_2_ saturation (SpO_2_) data were continuously collected and end-exercise values were defined as the average of values collected during the final 30 s of the bouts. Dyspnea and leg fatigue were assessed (see above) every 5 min.

### Open-circuit Spirometry

Expired O_2_ and carbon dioxide (CO_2_) concentrations and ventilatory volumes were measured breath by breath using open-circuit spirometry (Innocor, Innovision, Denmark). Airflow was measured by pneumotach using a differential pressure transducer (Innocor, Innovision, Denmark). Gases were sampled at a rate of 120 ml∙min^− 1^ and analysed by photoacoustic spectroscopy. The system was calibrated according to the manufacturer’s procedures using a 3-L syringe (Series 5530, Hans Rudolph Inc., Germany). A 12-lead ECG (Case 8000, Marquette GE, USA) was used to measure HR while pulse oximetry (Nonin 8500, Nonin Medical, INC, NH, USA) was used to measure SpO_2_. Blood samples were taken from an earlobe every minute during each of the three exercise tests and these samples were used to determine blood-lactate concentration ([lactate]) (Accu Check Softclix Pro Lancet, Accu Check, Australia).

### Quantification of V̇O_2_ kinetics

Breath-by-breath V̇O_2_ data from each CWR bout were initially examined to exclude errant breaths caused by coughing, swallowing, sighing, etc., and those values lying more than 4 SDs from the local mean were considered for removal. The breath-by-breath data were subsequently fit with an exponential curve to provide information regarding V̇O_2_ kinetics. We used a single exponential model without time delay with fitting window commencing at *t* = 0 s (i.e., the point at which the square-wave transition to the constant exercise work rate was made) to derive the MRT for the V̇O_2_ response. Specifically, we used a nonlinear least-squares algorithm as described in the following equation to fit the data:

$$ \mathrm{V}\dot {\mathrm{O}}_2\ (t)=\mathrm{V}\dot {\mathrm{O}}_{2\mathrm{base}}+\mathrm{A}\ \left(1-{\mathrm{e}}^{-\left(\mathrm{t}/\mathrm{MRT}\right)}\right) $$

where V̇O_2_ (*t*) is the absolute V̇O_2_ at a given time *t*, V̇O_2base_ represents the mean V̇O_2_ during the final 30 s of baseline cycling, A represents the amplitude of the response and MRT indicates the time that will be required for the response to reach ~ 63% of completion with no distinction made for the various phases of the response. An iterative process was used to minimise the sum of the squared errors between the fitted function and the observed values. We also determined the V̇O_2_ at minutes 3 and 6 of both CWR bouts so that we could calculate the increase in V̇O_2_ that occurred between these two time points and between minute 3 and exhaustion for CWR_70_. In lieu of modeling the various phases of the response, this index has been used to estimate the amplitude of the V̇O_2_ slow component that is present for CWR exercise above the lactate threshold [36].

### High-intensity interval training program

Each HIIT session involved work intervals performed at 70% of the peak work rate achieved on the incremental test followed by 60 s of recovery cycling at 35%. The overarching objective over the course of the eight-week intervention was to gradually increase the duration of the work intervals with the duration of the recovery intervals held constant. The increase was determined according to participant tolerance under the guidance of a therapist based on the ratings of leg fatigue and dyspnea. Each training session was limited to 45 min so when work-interval duration was increased in this manner, participants performed less repetitions. Each HIIT session comprised 5 min of warm-up cycling at 5–10 W followed by the work/recovery HIIT sequence. A five-minute active recovery period was allowed following the HIIT session. The first session was used to familiarize participants with the training protocol. During this familiarization session, subjects were limited to ≤15 min of performance of the high-intensity work intervals. As was the case with the incremental test (see above), throughout these training sessions, HR and SPO_2_% were measured continuously, blood [lactate] was assessed and dyspnea and leg-fatigue scores were monitored.

### Oxygen supplementation

During each HIIT training session, participants wore a nasal cannula through which either medical oxygen (100%) or ambient air was delivered continuously at 3 L∙min^− 1^. The former comprised the experimental condition (O2+) while the later represented the placebo treatment (AMB).

### Statistical analysis

Data are presented as mean ± SD. Between-group comparisons for baseline measurements were compared using an independent t-test. Changes in the physiological responses at peak exercise, upon completion of CWR_30_ and at the ‘isotime’ (i.e., completion time of the pre-training bout) for CWR_70_ were compared using time (pre-, post-training) x condition (O2+, AMB) repeated-measure analysis of variance. When a significant main effect was found, we examined estimated marginal means to determine its location. Statistical significance was accepted when *P* < 0.05. Data were analysed using SPSS v17.0 (SPSS Inc., IL).

## Results

**Fig. 1:**
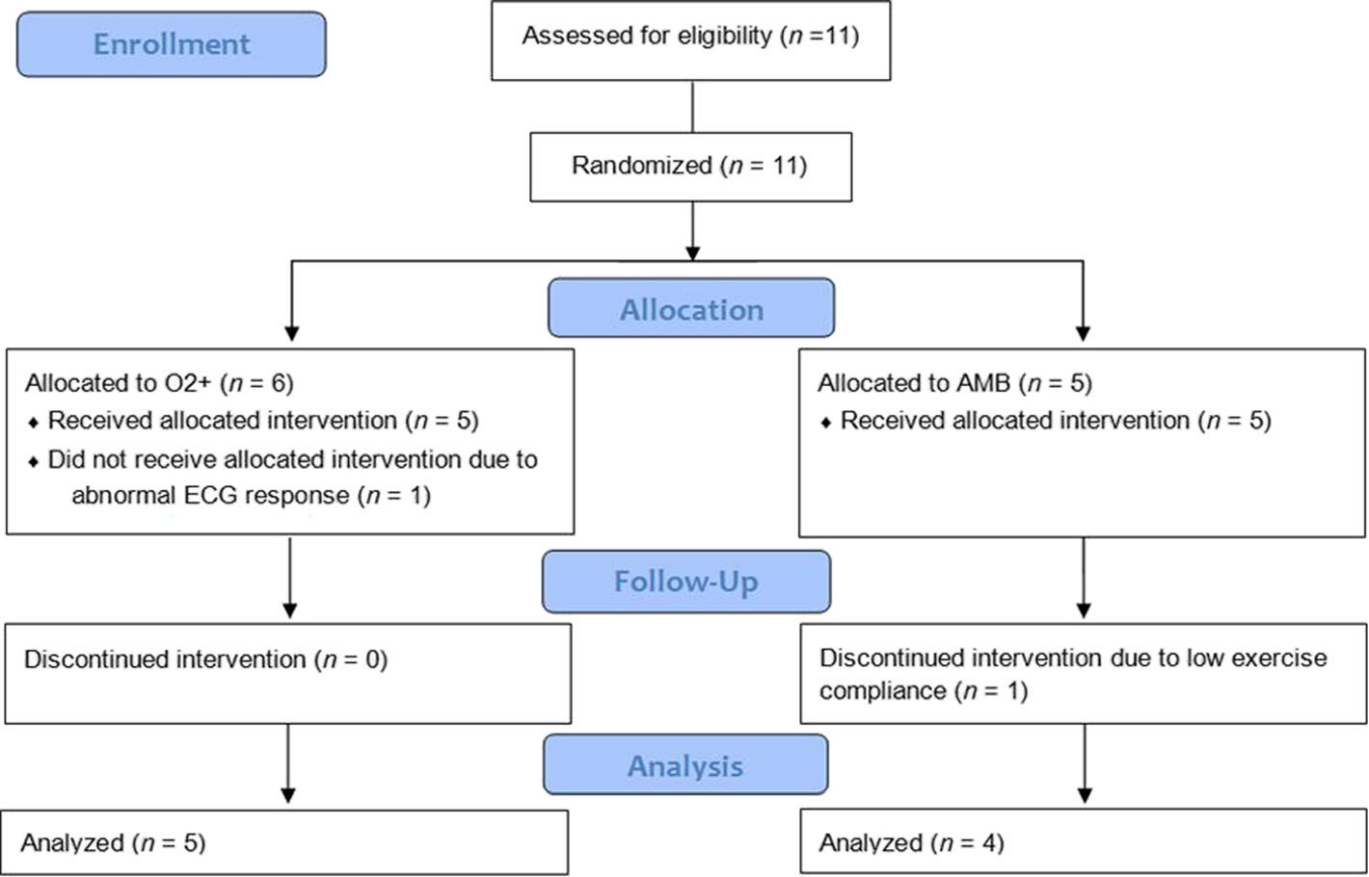
CONSORT diagram outlining the flow of subjects through the stages of the present investigation

A CONSORT diagram outlining the flow of subjects through the stages of the present investigation is provided in Fig. 1 and individual-subject anthropometric, pulmonary-function and incremental-test data at baseline are presented in Table 1. Two of the 11 recruited subjects (one from each group) did not complete the study. One subject was excluded due to low compliance with the training program and the other was excluded due to an abnormal ECG response. Training sessions had to be rescheduled for three subjects (O2+; *n* = 2) due to exacerbation of their CF diseased state. Three subjects in O2+ and three subjects in AMB were ‘respiratory limited’ by a low breathing reserve during the baseline incremental testing.

**Table 1:** Pre-training anthropometric, pulmonary-function and incremental-test data for the O2+ and AMB groups

	O2+	AMB	*P*
Age (y)	29 ± 7	27 ± 4	0.715
Height (cm)	169 ± 6	170 ± 10	0.894
Body mass (kg)	59 ± 7	64 ± 18	0.528
Lean body mass (kg)	50 ± 4	51 ± 15	0.845
FEV_1_ (% predicted)	58 ± 25	57 ± 36	0.943
FVC (% predicted)	84 ± 16	71 ± 30	0.456
DLCO (% predicted)	78 ± 12	83 ± 24	0.703
WR_peak_ (W)	117 ± 59	121 ± 71	0.927
V̇O_2peak_ (ml∙min^−1^)	1.60 ± 0.55	1.64 ± 0.71	0.921
V̇_Epeak_ (L∙min^−1^)	71 ± 27	63 ± 29	0.648

*FEV*_*1*_ Forced expiratory volume in 1 s, *FVC* Forced vital capacity, *DLCO* Diffusing capacity for carbon monoxide, *WR*_*peak*_ peak rate of work achieved on incremental cycling test, *V̇O*_*2peak*_ peak rate of oxygen uptake achieved on incremental cycling test, *V̇*_*Epeak*_ peak rate of minute ventilation achieved on incremental cycling test

### Pre-/post-training comparisons

#### Anthropometric and pulmonary-function measurements

There was no significant difference in body mass (*P* = 0.835) or lean body mass (*P* = 0.108) post compared to pre training in either group. However, a main effect of time was present for body-fat percentage (*P* = 0.021), which increased during the training intervention (O2+: pre, 21.1 ± 3.5%; post, 22.4 ± 3.8%; AMB: pre, 15.2 ± 6.7%; post, 15.9 ± 6.8%). There were no pre/post differences for any of the pulmonary-function measurements that were made (*P* > 0.05).

#### Incremental cycling test

The physiological responses during the incremental cycling test pre and post training are provided in Table 2. A main effect of time was observed for SpO_2_% (*P* < 0.05); however, no significant pre/post differences were observed for V̇O_2peak_ or peak HR for either group.

**Table 2:** Pre-/post-training measurements during the incremental cycling test to the limit of tolerance for the O2+ and AMB groups

	O2+		AMB		Time	Group x Time
	Pre	Post	Pre	Post	*P*	*P*
V̇O_2peak_ (ml^.^kg^− 1^LBM^.^min^− 1^)	32 ± 11	34 ± 11	32 ± 5	34 ± 11	0.387	0.963
WR_peak_ (W)	117 ± 59	131 ± 70	121 ± 71	137 ± 101	0.080	0.883
HR_peak_ (beats^.^min^− 1^)	167 ± 19	166 ± 19	159 ± 30	158 ± 22	0.866	0.929
Peak RER	1.1 ± 0.1	1.1 ± 0.0	1.1 ± 0.1	1.1 ± 0.1	0.933	0.783
V̇_Epeak_ (L^.^min^− 1^)	71 ± 27	72 ± 36	63 ± 29	61 ± 28	0.842	0.694
V_Tpeak_ (L)	1.6 ± 0.8	1.5 ± 0.9	1.6 ± 0.9	1.7 ± 1.3	0.757	0.672
Peak BR (breaths^.^min^− 1^)	49 ± 9	49 ± 9	44 ± 12	43 ± 13	0.908	0.706
Peak SpO_2_%	93 ± 4	90 ± 7*	93 ± 6	91 ± 5*	0.021	0.240
Peak [Lactate] (mmol^.^ L^− 1^)	6.2 ± 1.8	6.3 ± 2.5	6.4 ± 3.2	6.1 ± 2.2	0.762	0.704
Peak RPE	15.4 ± 2.5	15.6 ± 3.1	16.0 ± 1.4	16.5 ± 1.0	0.407	0.717
Peak dyspnea	6.6 ± 2.1	6.2 ± 2.4	6.0 ± 1.8	6.3 ± 1.0	0.879	0.515

Values are presented as mean ± SD. *V̇O*_*2peak*_ peak rate of oxygen uptake, *RER* Respiratory exchange ratio, *V̇*_*Epeak*_ peak rate of minute ventilation, *V*_*Tpeak*_ peak tidal volume, *BR* Breathing rate, *SpO*_*2*_ peripheral capillary O_2_ saturation, *[lactate]* blood lactate concentration, *RPE* Rating of perceived exertion, * = significantly different from pre-training value within group (*P* < 0.05)

#### Constant-work-rate tests

**Fig. 2:**
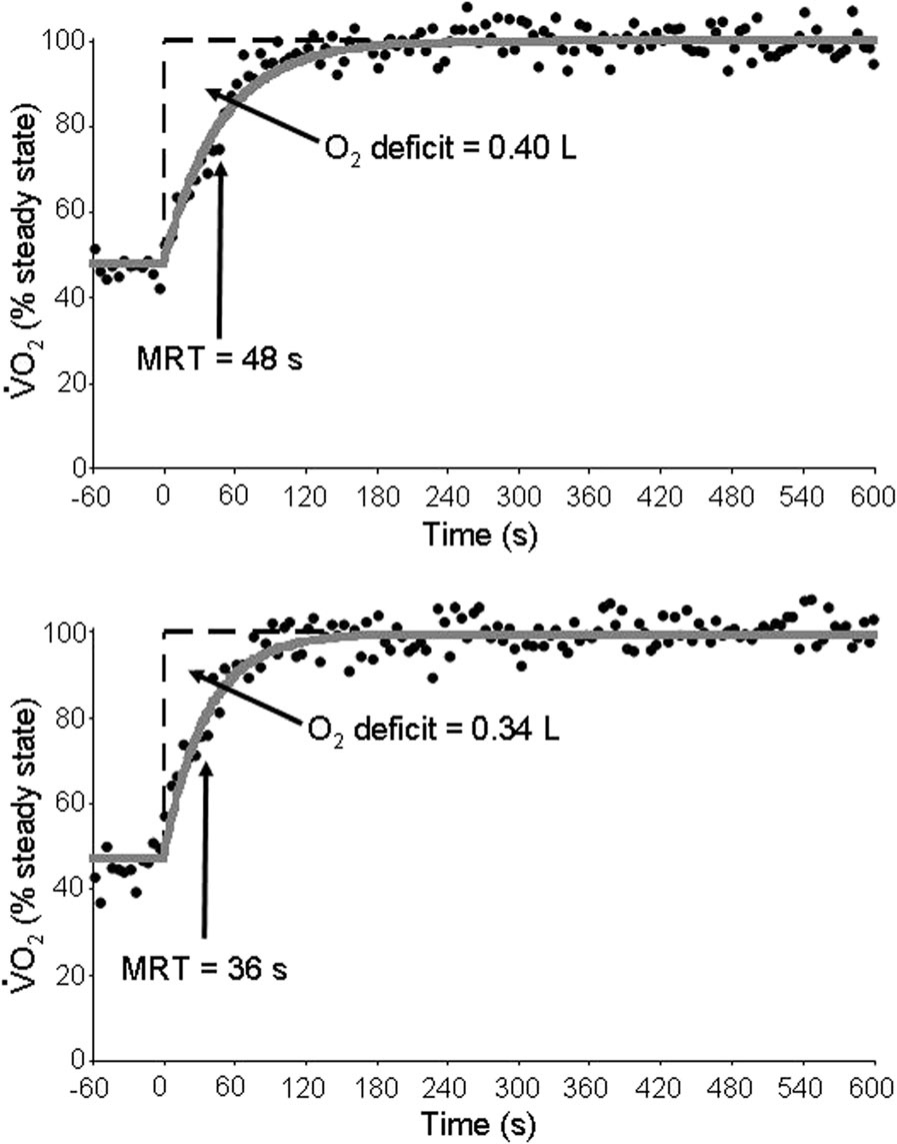
Rate of pulmonary oxygen uptake for a representative subject in the O2+ group before (top panel) and after (bottom panel) an eight-week training intervention comprising HIIT performed twice per week. The data were collected during unloaded cycling (time = − 60 to 0 s) followed by a square-wave increase to 30% of the subject’s peak work rate. Closed circles depict 5-s averages of breath-by-breath V̇O_2_ data while solid line depicts modelled fit. Notice the marked reduction in the V̇O_2_ MRT (i.e., the time taken for V̇O_2_ to reach ~ 63% of the steady-state amplitude) and consequent reduction in the O_2_ deficit indicated by the dashed line (O_2_ deficit = MRT x Amplitude)

The pre-/post-training measurements for constant-work-rate tests are presented in Table 3 (CWR_30_) and Table 4 (CWR_70_) and representative-subject data is provided in Fig. 2. For CWR_30_, a main effect of time was observed for the V̇O_2_ MRT (*P* = 0.000) indicating that HIIT accelerated low-intensity V̇O_2_ kinetics for these subjects. A main effect of time was also present for end blood [lactate] during CWR_30_ (*P* = 0.018) with a significant group-by-time interaction indicating that this was the case for O2+ (*P* = 0.009). During CWR_70_, HIIT increased time to limit of tolerance (*P* = 0.002) and decreased the blood [lactate] response (*P* = 0.003) with no group-by-time interaction observed for either variable (*P* = 0.143 and *P* = 0.102, respectively). Conversely, the V̇O_2_ MRT was unaltered by HIIT at the higher intensity of work (*P* = 0.168) although it was reduced in six of the nine subjects that were tested. At isotime, V̇_E_ and BR decreased with a significant group-by-time interaction indicating that this was the case for O2+ (*P* < 0.05).

**Table 3:** Pre-/post-training measurements during 10-min constant-work-rate test at 30% of peak work rate for O2+ and AMB groups

	O2+		AMB		Time	Group x Time
	Pre	Post	Pre	Post	*P*	*P*
Baseline V̇O_2_ (L^.^min^− 1^)	0.39 ± 0.06	0.40 ± 0.06	0.32 ± 0.08	0.36 ± 0.12	0.152	0.262
V̇O_2_ MRT (s)	44 ± 9	34 ± 11	45 ± 17	39 ± 14	0.000*	0.130
V̇O_2_ amplitude (L^.^min^− 1^)	0.54 ± 0.19	0.52 ± 0.21	0.59 ± 0.20	0.57 ± 0.24	0.391	0.968
∆V̇O_2 (6-3)_ (L^.^min^− 1^)	0.01 ± 0.06	0.03 ± 0.04	0.00 ± 0.03	0.04 ± 0.03	0.148	0.655
End V̇_E_ (L^.^min^− 1^)	32 ± 2	28 ± 5	29 ± 3	30 ± 4	0.119	0.024*
End V_T_ (L)	1.1 ± 0.5	1.1 ± 0.5	1.2 ± 0.5	1.1 ± 0.6	0.122	0.110
End BR (br^.^min^− 1^)	35 ± 11	30 ± 9	31 ± 13	31 ± 12	0.186	0.119
End SpO_2_%	94 ± 2	94 ± 2	95 ± 3	94 ± 3	0.407	0.079
End [lactate] (mmol^.^ L^− 1^)	2.9 ± 0.8	1.5 ± 0.3	1.6 ± 0.2	1.7 ± 0.6	0.018*	0.009*
End RPE	11.0 ± 1.4	9.8 ± 1.3	10.0 ± 2.9	10.8 ± 2.6	0.754	0.200
End dyspnea	2.2 ± 1.3	1.6 ± 1.5	2.3 ± 1.0	2.3 ± 1.7	0.461	0.461

Values are presented as mean ± SD. *MRT* Mean response time, *∆V̇O*_*2 (6-3)*_ change in V̇O_2_ from minute 3 to 6, V̇_E_, rate of minute ventilation, *V*_*T*_ tidal volume, *BR* Breathing rate, *SpO*_*2*_ peripheral capillary O_2_ saturation, *[lactate]* blood lactate concentration, *RPE* Rating of perceived exertion; * *P* < 0.05

**Table 4:** Pre-/post-training measurements during constant-work-rate test at 70% of peak work rate for O2+ and AMB groups

	O2+		AMB		Time	Group x Time
	Pre	Post	Pre	Post	*P*	*P*
Baseline V̇O_2_ (L^.^min^− 1^)	0.40 ± 0.07	0.41 ± 0.08	0.37 ± 0.06	0.37 ± 0.10	0.864	0.924
V̇O_2_ MRT (s)	61 ± 6	51 ± 14	64 ± 18	58 ± 29	0.168	0.699
V̇O_2_ amplitude (L^.^min^− 1^)	1.03 ± 0.41	0.99 ± 0.44	1.09 ± 0.58	1.06 ± 0.59	0.211	0.830
∆V̇O_2 (6-3)_ (L^.^min^− 1^)	0.11 ± 0.07	0.14 ± 0.14	0.11 ± 0.07	0.05 ± 0.08	0.625	0.068
Isotime V̇_E_ (L^.^min^− 1^)	58 ± 17	48 ± 12	46 ± 8	45 ± 10	0.006*	0.026*
Isotime V_T_ (L)	1.5 ± 0.7	1.4 ± 0.6	1.6 ± 0.9	1.5 ± 0.9	0.054	0.281
Isotime BR (br^.^min^− 1^)	42 ± 8	36 ± 7	36 ± 14	37 ± 16	0.041*	0.006*
Isotime SpO_2_%	90 ± 6	91 ± 4	90 ± 9	89 ± 7	0.668	0.413
Isotime [lactate] (mmol^.^ L^− 1^)	6.3 ± 1.4	4.5 ± 0.9	5.2 ± 1.4	4.5 ± 0.6	0.003*	0.102
Isotime RPE	14.4 ± 1.9	11.8 ± 1.8	13.8 ± 1.5	14.8 ± 2.9	0.213	0.018*
Isotime dyspnea	5.0 ± 1.9	4.0 ± 1.0	4.0 ± 1.2	4.5 ± 1.7	0.601	0.145
Limit of tolerance (min)	11 ± 2	25 ± 6	12 ± 6	18 ± 11	0.002*	0.143

Values are presented as mean ± SD. *MRT* Mean response time, *∆V̇O*_*2 (6-3)*_ change in V̇O_2_ from minute 3 to 6, V̇_E_, rate of minute ventilation, *V*_*T*_ Tidal volume, *BR* Breathing rate, *SpO*_*2*_ peripheral capillary O_2_ saturation saturation, *[lactate]* blood lactate concentration, *RPE* Rating of perceived exertion; * *P* < 0.05

#### High-intensity interval training

**Fig. 3:**
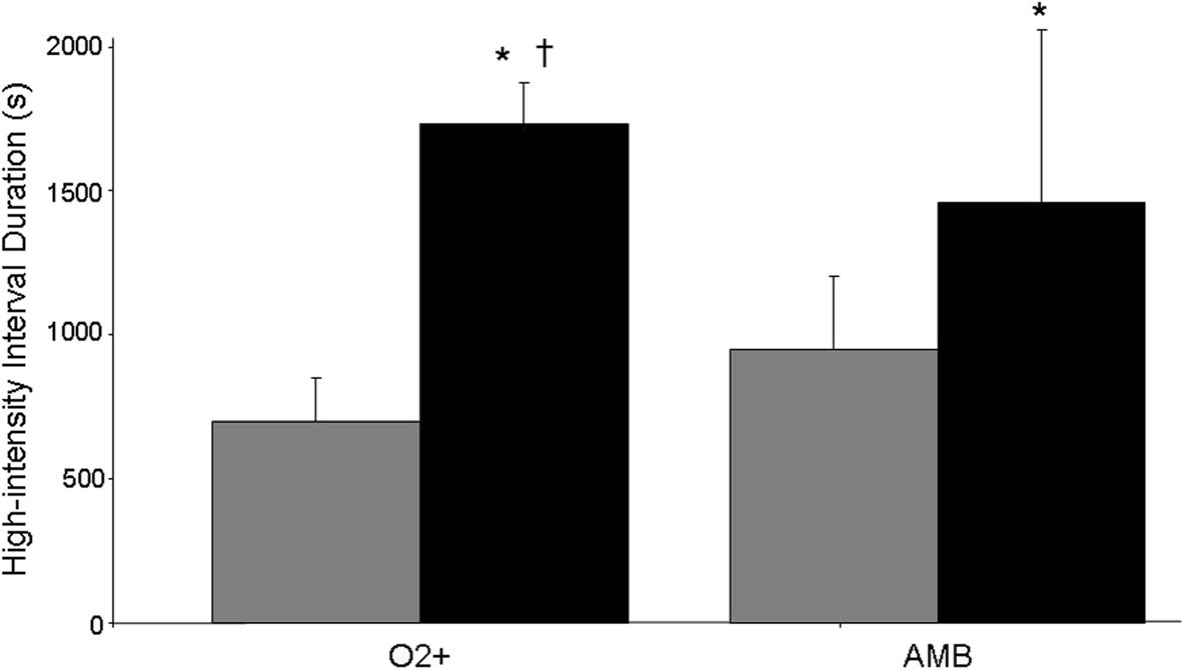
Group mean ± SD for work-interval duration during week one (gray bars) and week eight (black bars) for the O2+ and AMB groups. * *P* < 0.05 compared to week one value within group; † *P* < 0.05 compared to increase for AMB

The work rates for the work (O2+, 82 ± 42 W; AMB, 85 ± 50 W) and recovery (O2+, 41 ± 21 W; AMB, 42 ± 25 W) intervals were not significantly different between groups (*P* = 0.927). Over the course of the eight-week training intervention, both groups experienced a significant increase in work-interval duration during training sessions (*P* = 0.000); however, the change in O2+ was significantly greater than the change in AMB (*P* = 0.027; Fig. 3). Data exemplifying this progression for a representative subject in each group over the course of the intervention is provided in Table 5. Both groups also demonstrated a decreased blood [lactate] response during the final high-intensity interval of the sessions of week eight compared to week one (*P* = 0.014); however, in this case, there was no group-by-time interaction (O2+: pre, 4.3 ± 1.7 mmol^.^ L^− 1^; post, 3.0 ± 0.9 mmol^.^ L^− 1^; AMB: pre, 3.2 ± 0.2 mmol^.^ L^− 1^; post, 2.6 ± 0.5 mmol^.^ L^− 1^). Average SpO_2_% during the final 15 s of the final work interval during the training sessions ranged from 92 to 96% for O2+ and 92 to 94% for AMB. There was no significant difference in training SpO_2_% between groups over the eight-week training intervention.

**Table 5:** Progression in high-intensity work-interval duration for a representative subject from each group during the eight-week training intervention

SESSION #	O2 + (s)	AMB (s)
1	1029	1020
2	1157	960
3	1157	900
4	1200	1200
5	1350	972
6	1500	1044
7	1500	1620
8	1620	1800
9	1688	1800
10	1688	1929
11	1688	1929
12	1774	1929
13	1800	1929
14	1800	1288
15	1800	1714

## Discussion

The main original finding from this investigation is that an eight-week HIIT training program comprising 16 training sessions for individuals with CF shortened the V̇O_2_ MRT during constant-work-rate cycling at 30% of the participant’s peak work rate and lengthened the time to limit of tolerance during constant-work-rate cycling at 70%. These findings support our experimental hypotheses and are consistent with the contention that HIIT provides a potent training stimulus that can improve the ‘functional capacity’ of individuals with CF. We also found that the enhanced ability to perform HIIT afforded by O_2_ supplementation did not bring about a significant change in either of these effects. Although the potential for a type II error due to our limited sample size cannot be discounted (see study limitations below), the observation that an intervention that markedly increased the volume of exercise that these individuals were able to perform at the same intensity did not result in a greater training effect is consistent with the contention that the stimulus provided by HIIT is related to intensity and not volume [37].

In the present study, we had individuals with CF complete an eight-week training intervention comprising cycling bouts performed with ‘work intervals’ at work rates above the maximal sustainable pace interspersed with active recovery intervals of low-intensity cycling. High-intensity interval training of this type has long been a staple of athletic training [38]; however, in recent years, the efficacy and safety of HIIT for populations with cardiopulmonary pathologies have been explored and results have been promising [39, 40]. Consistent with prior observations, we found HIIT to be a safe, effective exercise strategy for individuals with CF. Specifically, with respect to the former, no adverse events were observed and with respect to the latter, HIIT shortened the V̇O_2_ MRT, which means that it accelerated V̇O_2_ kinetics. In all likelihood, this has important functional implications during activities of daily living for these individuals [13]. A faster V̇O_2_ response to a given increase in physical activity results in less metabolic perturbation (e.g., phosphocreatine degradation and blood [lactate] accumulation); hence, the cumulative positive effect of accelerated V̇O_2_ kinetics over the many submaximal transitions that comprise daily life has the potential to be considerable [14]. We also found a decrease in blood [lactate] accumulation and ventilation along with an increase in time to exhaustion during high-intensity exercise due to HIIT. While time to limit of tolerance during ‘unsustainable’ constant-work-rate exercise as a viable indicator of clinical significance can be questioned, this might represent additional evidence of the functional benefit that occurs due to HIIT. For example, in an official statement from the European Respiratory Society that reviewed the use of exercise testing in the evaluation of interventional efficacy, Puente-Maestu et al. state that time to exhaustion derived from a CWR is a particularly sensitive index of interventional change in respiratory diseases [19]. Previous research suggests that repeat-sprint training (SIT; i.e., HIIT performed with work intervals comprising all-out exercise) accelerates V̇O_2_ kinetics and improves high-intensity exercise tolerance in healthy subjects [41]; hence, our findings provide preliminary evidence that this observation might be extended to HIIT for the CF population.

The safety and efficacy of HIIT for individuals with CF has been established previously [28, 42]; however, with respect to the latter, outcome measures to determine HIIT’s beneficial effect were derived from maximal cardiopulmonary exercise testing (e.g., peak rates of V̇O_2_ and/or V̇_E_, ventilatory threshold) and did not include any that were related to V̇O_2_ kinetics. While this means of assessment represents the accepted way for testing ‘functional capacity,’ the degree to which an incremental test to limit of tolerance accurately reflects the capacity to meet the energetic demands of everyday life has been questioned [13]. Conversely, working at a submaximal rate of work (albeit not constant) provides a physical challenge more like that which is encountered on a daily basis. It has also been shown that CWR testing is better suited for detecting and quantifying changes in exercise capacity in COPD subjects [43]. Collectively, this implies that determining the beneficial effects of exercise training for individuals with CF should include an assessment like the CWR kinetics testing we employed. Interestingly, we also used incremental exercise to assess training-induced changes and found no effect on any of the parameters that we measured. The reason for this lack of coherence with previous research is unclear, but might represent differential effects secondary to disease progression of our CF subjects and/or the specific type of HIIT protocol that we employed.

It is well established that individuals with CF possess slower V̇O_2_ kinetics compared to healthy counterparts [13, 18, 26]. Generally speaking, V̇O_2_ kinetics can be rate limited by an upstream O_2_-transport limitation or a ‘metabolic inertia’ within the active muscle mitochondria [14] (i.e., a central or peripheral limitation, respectively). Although not without controversy [44], in healthy subjects during ‘normal’ exercise (e.g., walking, running, upright cycling), the preponderance of evidence suggests that V̇O_2_ kinetics is rate limited by metabolic inertia [14]. However, a reduced capacity for O_2_ delivery due to pathological changes, aging and/or environmental/experimental conditions (e.g., supine cycling, hypoxic inspiration) has the potential to shift the locus of limitation past a ‘tipping point’ thereby establishing O_2_ availability as the rate-limiting step [14]. It is intuitive to suggest that the pulmonary complications of CF would shift the site of limitation of V̇O_2_ kinetics past the tipping point into the O_2_-delivery-dependent zone, thereby explaining the slowed V̇O_2_ kinetics that has been observed for these individuals. However, Kusenbach et al. found that O_2_ supplementation (40%) that normalized O_2_ saturation in subjects with CF did not alter either V̇O_2_ kinetics or RER during cycling at submaximal work rates [25]. Furthermore, Saynor et al. found preserved cardiac function and central O_2_ delivery for children and adolescents with mild-to-moderate CF despite markedly slower V̇O_2_ kinetics during very-heavy cycling with the arterio-venous O_2_-content difference they measured during the initial 60 s of exercise significantly correlated with the V̇O_2_ MRT and phase II time constant (i.e., indices which indicate the rapidity of the V̇O_2_ response) [13]. Collectively, these findings suggest a limitation based on the ability of muscle mitochondria to extract/utilise the O_2_ that is delivered. Conversely, Hebestreit et al. observed an inverse relationship between SpO_2_ and the phase II V̇O_2_ time constant for subjects with CF during semi-supine cycling, which is consistent with rate limitation rooted in insufficient O_2_ delivery [26]. The reason(s) for these disparate findings is/are unclear, but might be related to the mode and/or intensity of the exercise challenge and/or the degree of disease progression for the subjects with CF. Although well beyond the scope of our investigation, it is interesting to note that the HIIT approach we employed which accelerated V̇O_2_ kinetics in individuals with CF did not increase V̇O_2peak_. Importantly, we did not assess central and peripheral function independently in this investigation; however, being that the maximal V̇O_2_ response would presumably be rate limited by central factors in these subjects [20] and given the propensity for HIIT to be particularly suited for inducing peripheral adaptations [45], our findings appear to support the contention that a peripherally-based factor was rate limiting V̇O_2_ kinetics in our CF subjects at least during the lower-intensity exercise challenge for which HIIT shortened the MRT. Moreover, during the higher-intensity exercise challenge, the ~ 90% increase in time to exhaustion elicited by HIIT was achieved despite unchanged SpO_2_ at the pre-training time to exhaustion. This finding might also be consistent with a HIIT-induced improvement in peripheral function because time to limit of tolerance was extended post training despite unchanged pathology-related pulmonary restrictions that have the potential to influence the limit of exercise tolerance for these individuals. However, more research is required to confirm this speculation using assessments that allow for differentiation of the two general potential locations of limitation/adaptation of oxidative function (i.e., central and peripheral).

In the present study, in addition to the effect of HIIT per se, we divided our cohort of nine CF subjects into a group that performed HIIT with O_2_ supplementation and another that did so while breathing ambient air. Our objectives were two-fold: first, to determine whether O_2_ supplementation would allow for longer high-intensity work intervals during HIIT; and second, to determine whether advantages afforded by O_2_ supplementation during HIIT would result in a greater training effect. We found that the increase in work-interval duration that occurred over the course of the eight-week HIIT intervention was significantly greater with O_2_ supplementation (see Fig. 2), which means that individuals with CF were able to perform longer periods of challenging work when they were breathing the hyperoxic inspirate. This is consistent with previous findings (see below); however, due to administrative challenges, we did not blind our test administrators to gas inspirate in the present study. Consequently, we cannot discount the possibility that administrator bias affected the decision making regarding the progressive increase in work-interval duration that was present over the course of the study. Importantly, these decisions were made based on the participant’s perceived responses (a subjective variable) which means that a bias might have affected the results. In addition to prior research (see below), future research should be designed to confirm that hyperoxic gas inspirate provided by administrators who are blinded to the intervention they are supplying improves the ability to progress high-intensity interval duration over the course of a study like the present one.

The increased ability to sustain high-intensity work intervals that we observed for individuals with CF during HIIT with hyperoxic gas inspiration is consistent with previous findings. For example, McKone et al. had subjects with moderate-to-severe CF perform submaximal cycling at 80% of their maximal work rate to the limit of tolerance both with and without O_2_ supplementation (39%) and found that the same end-exercise V̇O_2_, V̇_E_ and HR were achieved after an ~ 26% increase in cycling duration when subjects breathed the hyperoxic mixture [31]. This enhanced exercise tolerance occurred in association with an increase in arterial O_2_ saturation (~ 96% compared to ~ 86% in the control condition) with a significant relationship observed between the increase in exercise time and the amount of O_2_ desaturation during the control test [31]. This suggests that the ability for O_2_ supplementation to improve exercise tolerance depends on the degree of pathological compromise that is present for the individual. The reason(s) that O_2_ supplementation improves exercise tolerance for individuals with CF is unclear, but likely involves prolongation of the time taken for the individual to reach their maximum ventilatory capacity thereby reducing the sensation of dyspnea associated with the exercise challenge [31]. Specifically, maintenance of higher arterial PO_2_ during exercise results in both direct and indirect chemoreceptor inhibition, which reduces the ventilatory requirement [46]. Maintaining higher arterial saturation during exercise also has the potential to provide advantages at the periphery by allowing the same rate of mitochondrial respiration to be achieved with less perturbation of cellular phosphorylation state and redox potential. This would loosen a limitation to exercise tolerance that was present at the periphery.

Despite the marked amplification of the increase in work-interval duration that occurred over the course of our study, CF subjects that benefited from hyperoxic inspiration did not experience a greater reduction in the V̇O_2_ MRT (CWR_30_) or increase in time to exercise tolerance/decrease in blood [lactate] response (CWR_70_) compared to subjects inspiring ambient air. While this appears counterintuitive, a possible explanation is that the stimulus provided by HIIT is exclusively based on the overload provided by intensity; hence, it cannot be enhanced by an increase in volume. Support for this contention comes from a recent report that for individuals with metabolic syndrome, HIIT was equally effective for reducing disease severity when it was performed for 50 min per week compared to 114 [37]. Furthermore, for healthy individuals, a 10-week HIIT program (three sessions per week) improved V̇O_2max_ and decreased the O_2_ cost of submaximal work to a similar extent regardless of whether it comprised one 4-min work interval at 90% of maximal heart rate or four [47]. Indeed, it is this intensity dependence that is responsible for the time efficiency that is often touted as the major benefit of HIIT. Consequently, our findings cohere with this notion and suggest that the volume increase afforded by O_2_ supplementation during HIIT is not necessary to derive the benefits we observed for CF subjects from this form of training.

In addition to our finding of an improved capacity to transition to work intensities commensurate with those that occur during daily life, our findings might be important for individuals with CF because they imply that HIIT improves function irrespective of exercise volume and/or an intervention like supplemental O_2_ that might not be easily accessible to all individuals. However, with respect to the latter, it is important to note that our methodology (i.e., using supplemental O_2_ to increase the duration of work intervals with work rate held constant) does not allow us to rule out the possibility that O_2_ supplementation might be of benefit if intensity is allowed to fluctuate. For example, it is possible that an increase in work-interval work rate for the same duration of exercise might have resulted in a greater training effect. Alternatively, if O_2_ supplementation allows for an increased work rate during HIIT work intervals, individuals with CF might achieve the same training effect with a reduced exercise time commitment (for example, a 30-min HIIT session instead of 45). This would increase the likelihood of program adherence for a subject population at high risk for dropout [48]. Future research should explore these potential benefits of O_2_ supplementation with HIIT for individuals with CF.

There were a number of unexpected findings in our study. For example, a main effect for time indicated that body-fat percentage was increased in the entire cohort post compared to pre training. This might reflect the fact that we did not control energetic intake during the eight-week intervention. Participants were, therefore, able to voluntarily alter their ‘normal’ diet if they so desired and it appears that they did so by increasing energetic intake to a greater extent compared to the additional energetic outlay that was required for the training that was introduced into their daily regime. The end result was an energetic surplus that was stored as body fat. We also observed a significant main effect for time for measurement of SpO_2_ during incremental exercise to limit of tolerance which indicated greater O_2_ desaturation in the entire cohort post compared to pre training. This might be a function of the ‘trend’ for a main effect for peak work rate post compared to pre training; that is, participants were able to reach a higher absolute rate of work which required greater use of the O_2_ content of their blood. Although highly speculative (see above), this is consistent with our suggestion that the changes in function that were present post compared to pre training which would have allowed for this greater peak rate of work were located predominantly at the periphery (i.e. an increase in the ability to use O_2_ that is being delivered as opposed to an increase in the ability to deliver it).

Our study is not without significant limitations. In addition to the one regarding administrator bias (see above), a major one is the small sample size which increases the likelihood of a type II error. Consequently, this might explain why we found no effect of HIIT on the parameters assessed during incremental exercise and/or no effect of O_2_ supplementation on the benefits derived from HIIT during CWR exercise. The small sample size also had the potential to amplify the influence of differences that might have been present between participants prior to initiation of training that might have affected their response to the intervention and/or our statistical findings. We are also limited in our ability to provide greater insight into our observations regarding V̇O_2_ kinetics because we did not employ the more advanced modelling procedures that are necessary to isolate the various phases of the V̇O_2_ response. We chose the more rudimentary approach (i.e., fitting a single exponential curve to the entire V̇O_2_ response) because of concerns regarding the signal-to-noise ratio that is attainable when only one exercise transition is performed [49]. Future research exploring the influence of HIIT on V̇O_2_ kinetics and exercise tolerance should, therefore, be designed with larger sample sizes and pre/post CWR testing comprising multiple repetitions of the same exercise transition. Hopefully, the findings from our study will lead to research of this kind.

## Conclusions

In summary, we have shown that an eight-week training intervention comprising two HIIT sessions per week resulted in a reduced V̇O_2_ MRT during lower-intensity CWR cycling and increased time to exhaustion during higher-intensity CWR cycling for individuals with CF. We have also shown that despite a markedly greater increase in work-interval duration over the course of the training intervention, the training effect with compared to without O_2_ supplementation was similar. Collectively, these findings offer preliminary evidence that HIIT provides a time-efficient intensity-driven training stimulus that is appropriate for improving functional capacity in individuals with CF.

## Data Availability

The data collected during this investigation is available from the corresponding author upon request.

## Abbreviations

[lactate]: Blood lactate concentration
A: Amplitude
AMB: Group that inspired ambient air during training
BR: Breathing rate
CF: Cystic fibrosis
CO_2_: Carbon dioxide
CWR: Constant-work-rate exercise
DLCO: Diffusing capacity for carbon monoxide
FEV_1_: Forced expiratory volume in 1 s
FVC: Forced vital capacity
HIF1α: Hypoxia-inducible factor 1-alpha
HIIT: High-intensity interval training
HR: Heart rate
MRT: Mean response time
MVV: Maximal voluntary ventilation
O2+: Group that inspired supplemental oxygen during training
O_2_: Oxygen
RER: Respiratory exchange ratio
RPE: Rating of perceived exertion
SpO_2_: Peripheral capillary O_2_ saturation
V̇_E_: Rate of minute ventilation
V̇_Epeak_: Peak rate of minute ventilation
V̇O_2_: Rate of oxygen uptake
V̇O_2-base_: Baseline rate of oxygen uptake
V̇O_2peak_: Peak rate of oxygen uptake
V_T_: Tidal volume
WR_peak_: Peak rate of work on incremental cycling test
∆V̇O_2 (6-3)_: change in V̇O_2_ from minute 3 to 6

## References

[1] Hebestreit H, Kieser S, Junge S, Ballmann M, Hebestreit A, Schindler C, Schenk T, Posselt HG, Kriemler S (2010). Long-term effects of a partially supervised conditioning programme in cystic fibrosis. Eur Respir J.

[2] Rovedder PM, Flores J, Ziegler B, Casarotto F, Jaques P, Barreto SS, Dalcin PT (2014). Exercise programme in patients with cystic fibrosis: a randomized controlled trial. Respir Med.

[3] Kriemler S, Kieser S, Junge S, Ballmann M, Hebestreit A, Schindler C, Stüssi C, Hebestreit H (2013). Effect of supervised training on FEV1 in cystic fibrosis: a randomised controlled trial. J Cyst Fibros.

[4] Santana-Sosa E, Gonzalez-Saiz L, Groeneveld IF, Villa-Asensi JR, Barrio Gómez de Aguero MI, Fleck SJ, López-Mojares LM, Pérez M, Lucia A (2014). Benefits of combining inspiratory muscle with ‘whole muscle’ training in children with cystic fibrosis: a randomised controlled trial. Br J Sports Med.

[5] Hebestreit H, Kriemler S, Radtke T (2015). Exercise for all cystic fibrosis patients: is the evidence strengthening?. Curr Opin Pulm Med.

[6] van de Weert-van Leeuwen PB, Hulzebos HJ, Werkman MS, Michel S, Vijftigschild LA, van Meegen MA, van der Ent CK, Beekman JM, Arets HG (2014). Chronic inflammation and infection associate with a lower exercise training response in cystic fibrosis adolescents. Respir Med.

[7] Hommerding PX, Baptista RR, Makarewicz GT, Schindel CS, Donadio MV, Pinto LA, Marostica PJ (2015). Effects of an educational intervention of physical activity for children and adolescents with cystic fibrosis: a randomized controlled trial. Respir Care.

[8] Selvadurai HC, Blimkie CJ, Meyers N, Mellis CM, Cooper PJ, Van Asperen PP (2002). Randomized controlled study of in-hospital exercise training programs in children with cystic fibrosis. Pediatr Pulmonol.

[9] Schneiderman-Walker J, Pollock SL, Corey M, Wilkes DD, Canny GJ, Pedder L, Reisman JJ (2000). A randomized controlled trial of a 3-year home exercise program in cystic fibrosis. J Pediatr.

[10] Klijn PH, Oudshoorn A, van der Ent CK, van der Net J, Kimpen JL, Helders PJ (2004). Effects of anaerobic training in children with cystic fibrosis: a randomized controlled study. Chest..

[11] Pérez M, Groeneveld IF, Santana-Sosa E, Fuiza-Luces C, Gonzalez-Saiz L, Villa-Asensi JR, López-Mojares LM, Rubio M, Lucia A (2014). Aerobic fitness is associated with lower risk of hospitalisation in children with cystic fibrosis. Pediatr Pulmonol.

[12] Pianosi P, Leblanc J, Almudevar A (2005). Peak oxygen uptake and mortality in children with cystic fibrosis. Thorax..

[13] Saynor ZL, Barker AR, Oades PJ, Williams CA (2016). Impaired pulmonary V˙O2 kinetics in cystic fibrosis depend on exercise intensity. Med Sci Sports Exerc.

[14] Poole DC, Jones AM (2012). Oxygen uptake kinetics. Compr Physiol.

[15] Troosters T, Langer D, Vrijsen B, Segers J, Wouters K, Janssens W, Gosselink R, Decramer M, Dupont L (2009). Skeletal muscle weakness, exercise tolerance and physical activity in adults with cystic fibrosis. Eur Respir J.

[16] de Meer K, Gulmans VA, van Der Laag J (1999). Peripheral muscle weakness and exercise capacity in children with cystic fibrosis. Am J Respir Crit Care Med.

[17] de Meer K, Jeneson JA, Gulmans VA, van der Laag J, Berger R (1995). Efficiency of oxidative work performance of skeletal muscle in patients with cystic fibrosis. Thorax..

[18] Armeniakou E, Perpati G, Dimopoulos S, Roditis P, Avdikou M, Barouchos N, Dionisopoulou V, Nanas S (2015). Prolonged oxygen kinetics during constant workload submaximal exercise is associated with disease severity in adult subjects with cystic fibrosis. Respir Care.

[19] Puente-Maestu L, Palange P, Casaburi R, Laveneziana P, Maltais F, Neder JA, O'Donnell DE, Onorati P, Porszasz J, Rabinovich R, Rossiter HB, Singh S, Troosters T, Ward S (2016). Use of exercise testing in the evaluation of interventional efficacy: an official ERS statement. Eur Respir J.

[20] Pastré J, Prévotat A, Tardif C, Langlois C, Duhamel A, Wallaert B (2014). Determinants of exercise capacity in cystic fibrosis patients with mild-to-moderate lung disease. BMC Pulm Med.

[21] Shah AR, Gozal D, Keens TG (1998). Determinants of aerobic and anaerobic exercise performance in cystic fibrosis. Am J Respir Crit Care Med.

[22] Klijn PH, van der Net J, Kimpen JL, Helders PJ, van der Ent CK (2003). Longitudinal determinants of peak aerobic performance in children with cystic fibrosis. Chest..

[23] Labombarda F, Saloux E, Brouard J, Bergot E, Milliez P (2016). Heart involvement in cystic fibrosis: a specific cystic fibrosis-related myocardial changes?. Respir Med.

[24] Atlante A, Favia M, Bobba A, Guerra L, Casavola V, Reshkin SJ (2016). Characterization of mitochondrial function in cells with impaired cystic fibrosis transmembrane conductance regulator (CFTR) function. J Bioenerg Biomembr.

[25] Kusenbach G, Wieching R, Barker M, Hoffmann U, Essfeld D (1999). Effects of hyperoxia on oxygen uptake kinetics in cystic fibrosis patients as determined by pseudo-random binary sequence exercise. Eur J Appl Physiol Occup Physiol.

[26] Hebestreit H, Hebestreit A, Trusen A, Hughson RL (2005). Oxygen uptake kinetics are slowed in cystic fibrosis. Med Sci Sports Exerc.

[27] Lundby C, Jacobs RA (2016). Adaptations of skeletal muscle mitochondria to exercise training. Exp Physiol.

[28] Gruber W, Orenstein DM, Braumann KM, Beneke R (2014). Interval exercise training in cystic fibrosis -- effects on exercise capacity in severely affected adults. J Cyst Fibros.

[29] Nixon PA, Orenstein DM, Curtis SE, Ross EA (1990). Oxygen supplementation during exercise in cystic fibrosis. Am Rev Respir Dis.

[30] McKone EF, Barry SC, Fitzgerald MX, Gallagher CG (2005). Role of arterial hypoxemia and pulmonary mechanics in exercise limitation in adults with cystic fibrosis. J Appl Physiol.

[31] McKone EF, Barry SC, FitzGerald MX, Gallagher CG (2002). The role of supplemental oxygen during submaximal exercise in patients with cystic fibrosis. Eur Respir J.

[32] Jackson AS, Pollock ML (1978). Generalized equations for predicting body density of men. Br J Nutr.

[33] Miller MR, Hankinson J, Brusasco V, Burgos F, Casaburi R, Coates A, Crapo R, Enright P, van der Grinten CP, Gustafsson P, Jensen R, Johnson DC, MacIntyre N, McKay R, Navajas D, Pellegrino R, Viegi G, Wanger J, Pedersen OF (2005). Standardisation of spirometry. Eur Respir J.

[34] Macintyre N, Crapo RO, Viegi G, Johnson DC, van der Grinten CP, Brusasco V, Burgos F, Casaburi R, Coates A, Enright P, Gustafsson P, Hankinson J, Jensen R, McKay R, Miller MR, Navajas D, Pellegrino R, Wanger J, Pedersen OF (2005). Standardisation of the single-breath determination of carbon monoxide uptake in the lung. Eur Respir J.

[35] Hansen JE, Sue DY, Wasserman K (1984). Predicted values for clinical exercise testing. Am Rev Respir Dis.

[36] Whipp BJ, Wasserman K (1972). Oxygen uptake kinetics for various intensities of constant-load work. J Appl Physiol.

[37] Ramos JS, Dalleck LC, Borrani F, Beetham KS, Wallen MP, Mallard AR (2017). Low-volume high-intensity interval training is sufficient to ameliorate the severity of metabolic syndrome. Metab Syndr Relat Disord.

[38] Billat LV (2001). Interval training for performance: a scientific and empirical practice. Special recommendations for middle- and long-distance running. Part I: aerobic interval training. Sports Med.

[39] Ribeiro PA, Boidin M, Juneau M, Nigam A, Gayda M (2017). High-intensity interval training in patients with coronary heart disease: prescription models and perspectives. Ann Phys Rehabil Med.

[40] Kortianou EA, Nasis IG, Spetsioti ST, Daskalakis AM, Vogiatzis I (2010). Effectiveness of interval exercise training in patients with COPD. Cardiopulm Phys Ther J.

[41] Bailey SJ, Wilkerson DP, Dimenna FJ, Jones AM (2009). Influence of repeated sprint training on pulmonary O2 uptake and muscle deoxygenation kinetics in humans. J Appl Physiol.

[42] Hulzebos HJ, Snieder H, van der Et J, Helders PJ, Takken T (2011). High-intensity interval training in an adolescent with cystic fibrosis: a physiological perspective. Physiother Theory Pract.

[43] Borel B, Provencher S, Saey D, Maltais F (2013). Responsiveness of various exercise-testing protocols to therapeutic interventions in COPD. Pulm Med.

[44] Poole DC, Barstow TJ, McDonough P, Jones AM (2008). Control of oxygen uptake during exercise. Med Sci Sports Exerc.

[45] MacInnis MJ, Zacharewicz E, Martin BJ, Haikalis ME, Skelly LE, Tarnopolsky MA, Murphy RM, Gibala M (2017). Superior mitochondrial adaptations in human skeletal muscle after interval compared to continuous single-leg cycling matched for total work. J Physiol.

[46] Somfay A, Pórszász J, Lee SM, Casaburi R (2002). Effect of hyperoxia on gas exchange and lactate kinetics following exercise onset in nonhypoxemic COPD patients. Chest..

[47] Tjønna AE, Leinan IM, Bartnes AT, Jenssen BM, Gibala MJ, Winett RA, Wisløff U (2013). Low- and high-volume of intensive endurance training significantly improves maximal oxygen uptake after 10-weeks of training in healthy men. PLoS One.

[48] Prasad SA, Cerny FJ (2002). Factors that influence adherence to exercise and their effectiveness: application to cystic fibrosis. Pediatr Pulmonol.

[49] Lamarra N, Whipp BJ, Ward SA, Wasserman K (1987). Effect of interbreath fluctuations on characterizing exercise gas exchange kinetics. J Appl Physiol.

